# Ampullectomy of an unusual dysplastic lesion after deroofing of a type III choledochocele

**DOI:** 10.1016/j.vgie.2024.11.001

**Published:** 2024-11-06

**Authors:** Elena De Cristofaro, Claire Michoud, Jérôme Rivory, Pierre Lafeuille, Alexandru Lupu, Alice Burgevin, Mathieu Pioche

**Affiliations:** 1 Gastroenterology Unit, Department of Systems Medicine, University of Rome Tor Vergata, Rome, Italy; 2 Gastroenterology and Endoscopy Unit, Edouard Herriot Hospital, Hospices Civils de Lyon, Lyon, France

A type III choledochocele is a rare congenital cystic dilation of the intraduodenal portion of the main bile duct, representing 5% of all choledochal cysts according to the Todani classification.^1^ The exact cause of choledochoceles is not entirely understood, but it is thought to result from an abnormal development of the pancreaticobiliary junction, leading to bile duct dilation. When the choledochocele is symptomatic, the incidence of carcinoma is estimated to be 2.5%, and this risk increases with age.^2^

Endoscopic treatment is the preferred minimally invasive approach, involving cystic mass removal and sphincterotomy.^3^^,^^4^

We present a case of a type III choledochocele with a suspicious polypoid-like lesion managed endoscopically (Video 1, available online at www.videogie.org), with the minimally invasive approach.

A 76-year-old woman with a history of diabetes, obesity, and hypertension was referred to our center for a second opinion regarding a suspected choledochocele that was incidentally discovered on magnetic resonance imaging. Initial EUS revealed a Todani III choledochocele with a polypoid-like lesion within the collection (Fig. 1). The major papilla orifice was not visualized, precluding ERCP including with a cholangioscope. Thus, an EUS-guided endoscopic mucosal resection (EMR) with a snare was performed. This technique allowed for real-time visualization of the snare, facilitating deroofing and enabling the exposure and biopsy of the suspected villous lesion (Fig. 2). Histologic examination revealed low-grade dysplasia (Fig. 3).

**Figure 1:**
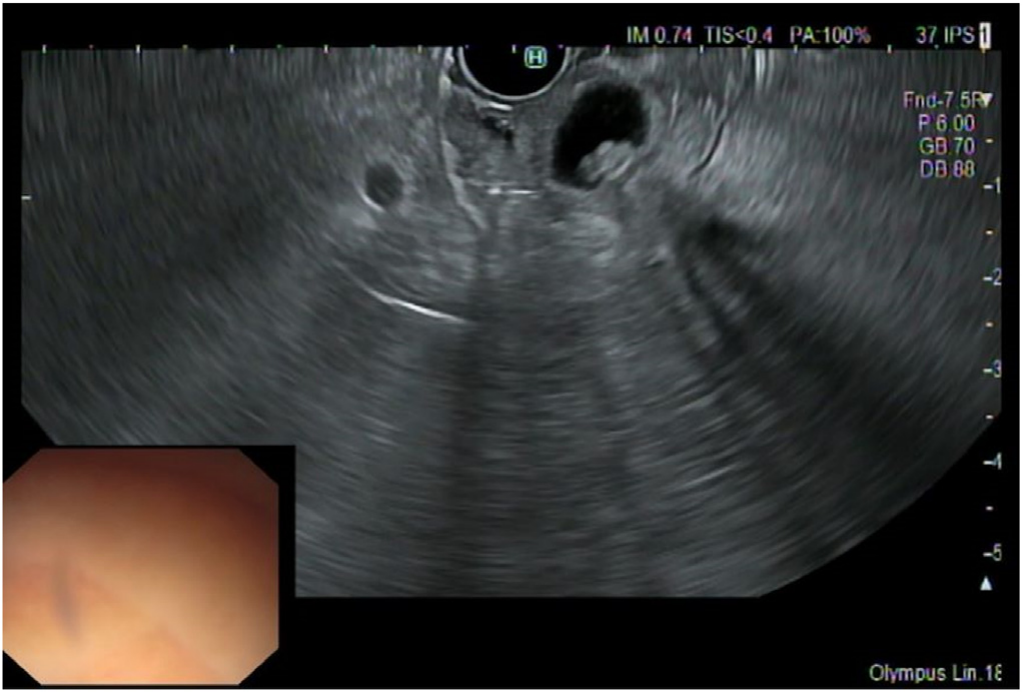
Todani III choledochocele with a polypoid-like lesion within the collection revealed at EUS.

**Figure 2:**
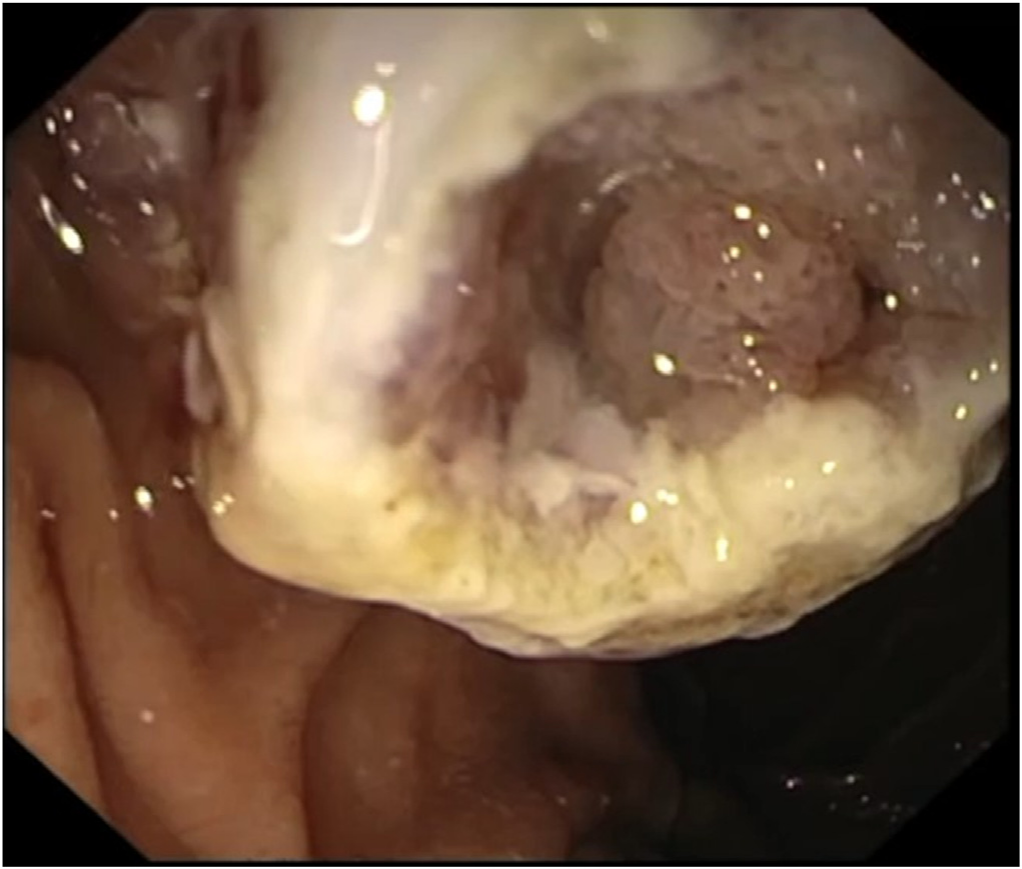
Suspicious villous lesion suggestive of dysplastic degeneration visible after EUS-guided EMR.

**Figure 3:**
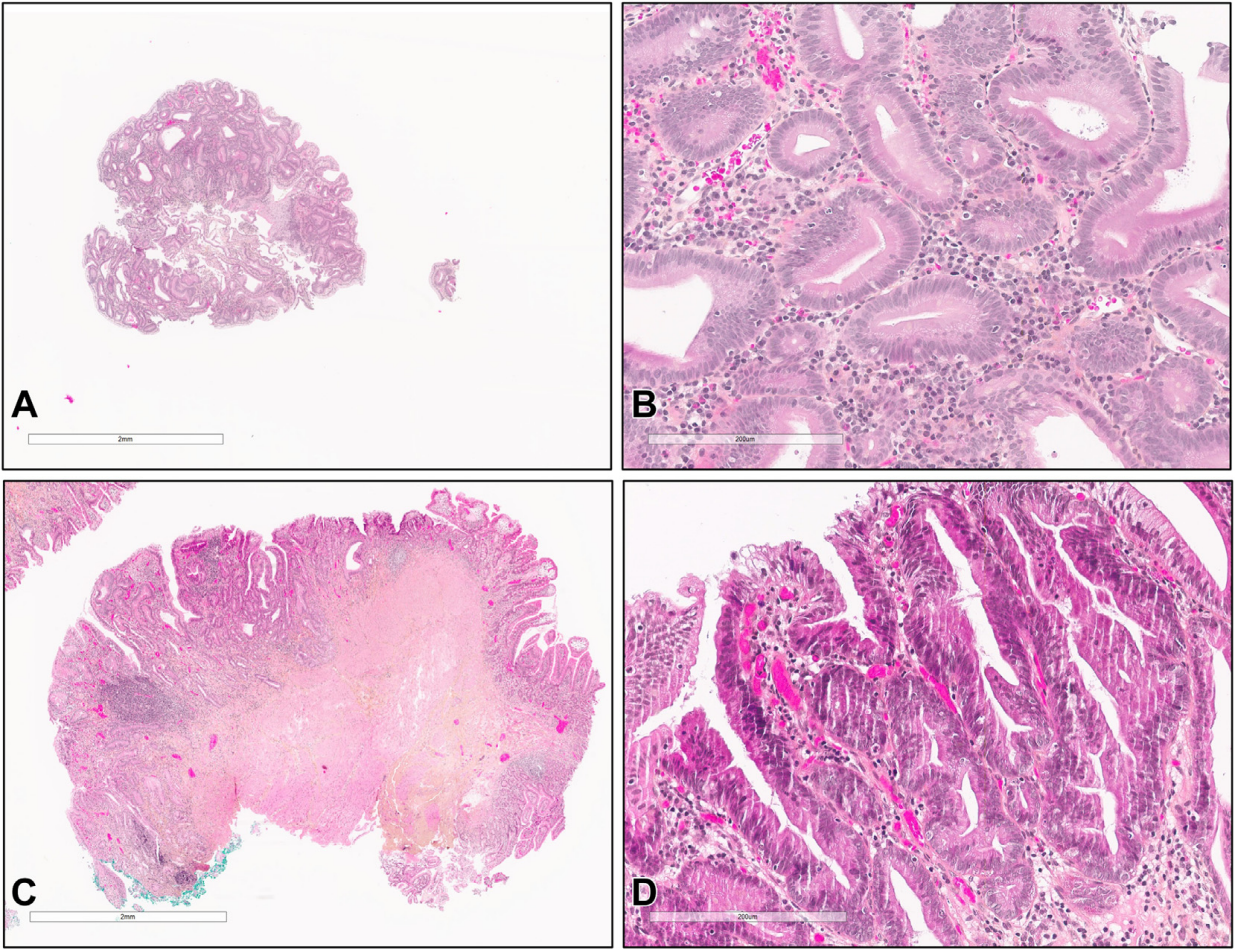
Histologic characterization of the dysplastic lesion obtained from the EMR and the ampullectomy resection, stained with hematoxylin and eosin, shown at low power (**A,** 2×; panel **C,** 4×) and high power (**B,** 20×; **D,** 20×) magnification. Glandular disorganization was observed at low power magnification, whereas cellular atypia, nuclear stratification, and frequent mitoses were evident at high power magnification.

After a multidisciplinary discussion, ampullectomy was recommended to minimize the risk, considering the potential for malignant progression. Preliminary EUS confirmed the absence of ductal invasion. The endoscopic examination revealed an adenomatous papilla with the lesion confined to the area of the previous choledochocele. Complete endoscopic resection of the papilla and the base of the choledochocele was achieved through a piecemeal approach, removing the lesion in 3 fragments. Prevention of post-ampullectomy pancreatitis was ensured by intrarectal indometacine administration and pancreatic stenting with a 5F × 5-cm plastic stent. Final histology confirmed low-grade dysplasia residue (Fig. 3). No pancreatitis or bleeding occurred after the resection.

In this case, an EUS-guided EMR enabled targeted biopsies of the suspected lesion in a very rare condition. This endoscopic approach aimed to achieve a step-up approach with justified complete resection after confirmation of dysplasia. In addition, it offers a cost-effective and time-saving alternative to other endoscopic procedures (eg, cholangioscopy). This advantage is particularly relevant when biliary cannulation during ERCP proves challenging.

## Disclosure

The authors disclosed no financial relationships.

## Supplementary data

Video 1 Type III choledochocele with a suspicious polypoid-like lesion managed endoscopically.
